# The Silent Complication: Auditory Dysfunction in Pediatric Patients with Type 1 Diabetes

**DOI:** 10.3390/jcm15020889

**Published:** 2026-01-22

**Authors:** Sara Shefa, Aleksandra Głębocka, Tetiana Zinyk, Karolina Dorobisz

**Affiliations:** 1 Student Scientific Association of Otolaryngology, Head and Neck Surgery, Wroclaw Medical University, 213 Borowska St., 50-556 Wrocław, Poland; aleksandra.glebocka@student.umw.edu.pl (A.G.); tetiana.zinyk@student.umw.edu.pl (T.Z.); 2 Department of Otolaryngology, Head and Neck Surgery, Wroclaw Medical University, 50-556 Wrocław, Poland

**Keywords:** type 1 diabetes, hearing loss, sensorineural hearing loss, auditory dysfunction

## Abstract

Diabetes is known to affect metabolic, vascular, and nervous systems, although its influence on auditory function in children remains poorly defined. Understanding this association is essential due to its implications for cognitive, language, and social development. Numerous studies have found that children with Type 1 Diabetes Mellitus (T1DM) exhibit higher hearing thresholds at high frequencies (4000–8000 Hz) and lower speech understanding scores compared to healthy controls. Poor glycemic control and longer disease duration are consistently associated with worse auditory outcomes. The proposed mechanisms include microangiopathy and diabetic neuropathy affecting the auditory pathway. Many affected children do not report noticeable auditory symptoms, indicating a risk of underdiagnosis. Early identification is crucial, as hearing difficulties in children may be related to underlying diabetic conditions and are likely associated with poor glycemic control. Regular audiometric screening should be incorporated into the routine care of pediatric diabetes patients to identify hearing deficits before they affect communication and cognitive development.

## 1. Introduction

Data from 2021 show that hearing disorders affect more than 97 million children and young adults up to the age of 20 [1]. Hearing loss in children significantly reduces their quality of life. It may contribute to delays in speech and language development and, in the long term, negatively affects social life and self-esteem, thereby increasing the risk of depression [2]. Hearing loss can be classified as congenital, which is genetically determined or occurs during fetal life due to the influence of teratogens, or acquired, which develops after birth because of external factors such as inflammatory changes, injuries, ototoxic drugs, or metabolic diseases. Sensorineural hearing loss (SNHL) is associated with changes in the central nervous system, the cochlear nerve, or the cochlea itself [3].

According to 2021 data, approximately 1.5 million children and adolescents under the age of 20 worldwide are living with Type 1 Diabetes Mellitus (T1DM) [4]. T1DM is a metabolic disease characterized by hyperglycemia caused by impaired insulin secretion, resulting from the apoptosis of pancreatic islet beta cells due to autoimmune processes [3]. While complications such as retinopathy, nephropathy, and peripheral neuropathy are well-known, hearing loss is often overlooked, as evidenced by the relatively limited number of studies on the subject.

The aim of this paper is to discuss the current knowledge regarding the impact of T1DM on hearing disorders in the pediatric population to draw greater attention to this issue.

## 2. Methods

A narrative review of peer-reviewed literature was conducted to explore the association between T1DM and auditory dysfunction in the pediatric population. The literature search was performed using the PubMed and Scopus databases using keywords: “Type 1 diabetes”, “pediatric hearing loss”, “audiometry”, and “sensorineural hearing loss”. Articles published between 2010 and 2025 were considered.

## 3. Results

### 3.1. Pathophysiology and Epidemiology of Hearing Loss in Children with T1DM

T1DM leads to the development of angiopathy—damage to small and large blood vessels. Microvascular changes are particularly important, as they may impair the blood supply to the organ of Corti in the inner ear [5]. Chronic hyperglycemia causes thickening of the vascular basement membrane and endothelial damage, which has also been confirmed in experimental studies of the vessels of the labyrinth [6]. In mice with T1DM, a significant reduction in the thickness of the stria vascularis has been observed, along with the loss of intermediate cells in this structure and deformation of the cochlear capillaries [6]. Such changes lead to hypoxia of cochlear structures and disturbances in the ionic balance of the endolymph, resulting in the gradual degeneration of auditory hair cells [7]. Therefore, patients with T1DM (including children and adolescents) exhibit a significantly higher incidence of hearing loss compared to the general population [8]. These observations indicate that diabetic angiopathy, through microcirculatory insufficiency within the ear, plays a significant role in the pathogenesis of hearing disorders in patients with T1DM [5,8]. Type 1 diabetes promotes the development of peripheral and autonomic neuropathy, which can affect the auditory system. In patients with T1DM, prolonged brainstem auditory evoked response (ABR) wave latencies are observed, indicating impaired nerve conduction in the auditory pathways [8,9]. In the Akita mouse model of T1DM, significant loss of spiral ganglion neurons and afferent fibers was observed, as well as increased activation of apoptotic caspases in auditory neurons [6,7]. Such degenerative changes within the spiral ganglion and auditory nerve disrupt the transmission of acoustic impulses, leading to increased hearing deficits. Additionally, atrophy of spiral neurons and demyelination of the auditory nerve have been reported in patients with diabetes [5]. This mechanism resembles the early phase of peripheral neuropathy—demyelination of nerve fibers—which may further impair hearing function. Considering these findings, diabetic neuropathy significantly contributes to the pathogenesis of hearing disorders in T1DM [6,10]. From 1990 to 2019, there was a 39.4% increase in the incidence of diabetes among pediatric patients [11], while the number of hearing loss cases among children and adolescents increased from approximately 79.9 million in 1990 to 97.8 million in 2021 [1]. According to the World Health Organization (WHO), the number of individuals experiencing hearing loss will increase to 700 million by 2050 [12]. Children with T1DM are increasingly recognized as a population at risk for subclinical and clinical hearing impairment. A study involving 26 children with T1DM [mean age 12.5 years] found that those with longer disease duration had significantly lower speech discrimination scores, indicating difficulties in processing speech sounds despite generally normal hearing thresholds. This finding suggests that auditory processing deficits may develop over time in children with T1DM, independently of glycemic control [13]. In a separate clinical study conducted between 2011 and 2012, involving 84 children and adolescents aged 6 to 18 years with T1DM, the prevalence of SNHL was reported to be 14.3% [14]. A meta-analysis including 15 studies reported that seven studies predominantly examined pediatric populations (322 children with T1DM and 282 healthy controls). In the pooled prevalence analysis of hearing loss (four studies; 252 individuals with T1DM and 253 controls), T1DM was associated with markedly higher odds of hearing loss (OR 49.08, 95% CI 12.03–200.31). However, this unusually large estimate is highly imprecise (very wide CI) and should be interpreted cautiously, as small sample sizes and sparse-event data can inflate effect estimates. Moreover, substantial between-study heterogeneity was reported for several pooled audiologic outcomes (e.g., pure-tone thresholds at 4000 Hz: I^2^ = 95%; ABR latency measures: I^2^ up to 86%), reflecting clinical and methodological variability in hearing assessment protocols and outcome definitions. Study quality (Newcastle–Ottawa Scale) ranged from 6 to 8 points, and while formal assessments did not suggest significant publication bias, it cannot be excluded given the small number of contributing studies for some outcomes. Overall, the findings support a possible association, but the magnitude remains uncertain and should be considered hypothesis-generating [9]. Another meta-analysis, which included nine studies assessing the prevalence of hearing loss (HL) in pediatric and adolescent populations with T1DM, reported that HL prevalence among diabetic patients ranged from 5.17% to 48%, compared to 0–40% in control groups. In studies focusing specifically on pediatric populations, the reported prevalence was between 7.5% and 33% in T1DM patients, while no cases were observed in control groups. These findings suggest that even in younger individuals, T1DM may be associated with a significantly increased risk of hearing impairment [8].

### 3.2. Hearing Assessment, Impact of Disease Duration and Glycemic Control

Effective management of hearing difficulties in children requires careful and age-appropriate evaluation. In the pediatric population, this task is particularly challenging, as developmental, cognitive, and communication abilities may limit a child’s ability to recognize or report auditory difficulties [15]. Proper auditory perception is important in social interactions and allows individuals to express thoughts and emotions, forming the basis of everyday communication [16]. Many children with early hearing loss do not report their symptoms and this defect is mostly unnoticed [17]. Ongoing cognitive difficulties can negatively affect the quality of life, learning, and overall development of children with T1DM [15,18]. Mild or unilateral hearing impairment has been shown to cause significant delays in vocabulary development, phonological awareness, and articulation. Research by Lieu et al. demonstrated that children with unilateral hearing loss exhibited significantly lower scores in oral comprehension (mean 91 vs. 98, *p* = 0.003), expression (94 vs. 101, *p* = 0.007), and composite language abilities (90 vs. 99, *p* < 0.001) compared to their siblings with normal hearing [19]. Moreover, these children were over four times more likely to require individualized education plans and more than twice as likely to receive speech–language therapy (43.9% vs. 16.7%), further highlighting the direct impact of hearing dysfunction on academic achievements [19]. In children with chronic illnesses such as T1DM, who already experience psychosocial stress, treatment fatigue, and educational disruption [18,20], coexisting hearing loss can further increase the risk of isolation, low self-esteem, and behavioral challenges [21,22].

Patients with poor glycemic control more frequently show abnormalities in audiological tests compared to the control group [23]. Numerous studies have demonstrated that elevated HbA1c levels are associated with higher hearing thresholds [24,25,26,27] and abnormal brainstem auditory evoked potentials (BAEP) [28]. Furthermore, the development of cochlear abnormalities has been linked to poor metabolic control [17]. The duration of T1DM is also an important risk factor for SNHL in children. The study by Treviño-González et al. demonstrated that both longer disease duration and poor glycemic control were significantly correlated with a higher risk of hearing loss [14]. Other authors have also concluded that longer disease duration is associated with an increased risk of hearing impairment in these patients [14,28]. Multiple studies indicate that diabetes may contribute to hearing impairment in children, particularly affecting high-frequency hearing [13,24,25,26]. In a retrospective clinical study, Yorulmaz et al. demonstrated that children with T1DM lasting 5 years or longer had significantly higher hearing thresholds at 4000 Hz and 8000 Hz compared to those with the disease for less than 5 years (*p* < 0.05) [13]. However, findings remain inconsistent regarding the lateralization of hearing loss; some studies report a predominance on the left side [26], while others have observed a greater impairment on the right [14] (Table 1).

## 4. Discussion

### 4.1. The Need for Early Audiological Diagnosis

Children with T1DM are at increased risk of developing microvascular complications that can affect auditory function, including damage to the cochlear vasculature and auditory pathways [30,31,32]. Several studies included in this review [8,9,33] consistently demonstrate that auditory dysfunction in children with T1DM often presents without clinical symptoms, without overt symptoms or complaints. As previously shown, strong correlation has been observed between poor glycemic control, elevated HbA1c levels, longer disease duration, and the progressive worsening of auditory thresholds, particularly at high frequencies [9,21,26]. These findings underscore the importance of early and routine audiological evaluations, even in asymptomatic children, to identify at-risk individuals before irreversible auditory or developmental consequences emerge. Early detection of hearing impairment allows for timely and targeted interventions—such as speech therapy, hearing aids, or specialized educational support—which can significantly mitigate the adverse effects on language acquisition, academic performance, and psychosocial well-being. Implementing regular hearing assessments as part of standard diabetes management, particularly in children with suboptimal glycemic control or longer disease duration, is a proactive approach that supports both clinical and developmental outcomes. This highlights the need to shift from a reactive to a proactive model of care, where hearing screening is conducted routinely rather than in response to reported deficits. Early audiological diagnosis not only helps prevent cumulative developmental delays but also contributes to improved long-term quality of life in pediatric T1DM patients [33,34]. Despite the growing evidence, hearing screening is still not integrated into most pediatric diabetes care guidelines, highlighting a critical gap in routine management.

### 4.2. Integrating Hearing Assessment into Pediatric Diabetes Care

Given the emerging evidence linking T1DM to early-onset, often subclinical auditory dysfunction, there is a compelling need to incorporate structured hearing evaluations into routine pediatric diabetes management. Despite increasing data supporting this association, hearing assessment is not currently embedded within standard clinical guidelines, representing a significant gap in comprehensive monitoring and early detection.

A practical and effective approach would involve the implementation of periodic audiometric screening, particularly for children with longer disease duration or with persistently elevated HbA1c levels. Screening could be conducted on an annual or biennial basis, with initial baseline testing shortly after diagnosis, followed by routine follow-up assessments depending on individual risk factors and glycemic control history. For early identification of subclinical cochlear involvement, extended high-frequency audiometry is more sensitive than conventional audiometry and is recommended as a primary screening tool in cooperative, older children [26,35]. In younger children, where behavioral testing may be unreliable, otoacoustic emissions (OAEs)—particularly transient evoked OAEs (TEOAEs)—are commonly recommended due to their objectivity, frequency specificity, and capacity to detect cochlear pathology before symptom onset [16]. TEOAE amplitude reductions have been reported in children with T1DM and may reflect early cochlear dysfunction. However, findings across studies have been variable, and the sensitivity of OAEs alone may be insufficient. In a recent cross-sectional study, Braite et al. found that children with T1DM more frequently reported memory (*p* = 0.002) and attention difficulties (*p* = 0.021) compared to healthy controls. Furthermore, prolonged latencies were observed in BAEP, specifically in wave III in the right (*p* = 0.017) and left (*p* = 0.019) ears, as well as in wave V in the left ear (*p* = 0.001) [29]. Another study demonstrated that elevated HbA1c levels were associated with increased latencies of both CAEPs and P300 responses [36]. These findings suggest that poor glycemic control may slow central auditory signal transmission and contribute to early cognitive dysfunction in children with type 1 diabetes. For this reason, auditory brainstem response (ABR) testing can be a valuable adjunct. Prolonged wave I or I–V interpeak intervals observed in pediatric T1DM patients may indicate early auditory neuropathy, which standard audiometry and OAEs may fail to detect. Notably, cochleovestibular symptoms are often absent in this population, further justifying the use of neurophysiological tests such as ABR to uncover subclinical pathology. Rance et al. [37] provided compelling evidence for the need for comprehensive, multimodal assessment. In school-aged children with T1DM, their study revealed significantly elevated hearing thresholds, reduced distortion product OAE (DPOAE) amplitudes, and prolonged auditory brainstem conduction times, despite normal results on standard pure-tone audiometry. These children also showed impaired speech perception in noisy environments, which correlated with auditory brainstem dysfunction. The authors concluded that functional hearing deficits capable of compromising communication and academic success are common in children with T1DM and that standard audiometric screening is inadequate for early detection [25].

Furthermore, early identification of high-frequency hearing loss may serve as a valuable clinical indicator, enabling interventions that could preserve auditory function and stabilize cognitive and academic performance [9,34,36]. Diabetic children with an extended high-frequency (EHF) mean hearing threshold exceeding 15 dB HL should be closely monitored, particularly in relation to glycemic control, as elevated HbA1c levels are associated with worsening auditory outcomes [25,26,33]. This dual focus—on both auditory surveillance and improved glycemic regulation—offers the potential to improve long-term outcomes across cognitive, linguistic, and academic domains of child development.

### 4.3. Evidence Versus Expert Opinion in Auditory Screening Recommendations for T1DM

The medical community widely endorses holistic care in T1DM; however, a clear discrepancy remains between the strength of evidence linking T1DM to auditory dysfunction and the explicit inclusion of auditory screening within major clinical practice guidelines. In guideline development frameworks such as those used by the ADA and ISPAD, recommendations are graded according to evidence quality, ranging from Grade A (high-quality randomized controlled trials) to Grade E (expert consensus in the absence of definitive data). The association between T1DM and hearing loss itself is supported by Grade B evidence, with numerous well-conducted cross-sectional and case–control studies demonstrating an increased prevalence of auditory impairment in children with T1DM, alongside plausible and well-substantiated pathophysiological mechanisms including microangiopathy and neuropathy. In contrast, recommendations for routine, periodic audiological screening remain largely grounded in Grade E expert opinion, as no large-scale randomized trials have directly shown that systematic screening improves long-term educational, communicative, or quality-of-life outcomes compared with symptom-driven referral. This distinction is reflected in current guidelines: ISPAD and ADA documents acknowledge hearing impairment as a potential comorbidity but do not mandate proactive audiological surveillance with the same specificity or evidentiary grading applied to retinopathy, nephropathy, or neuropathy screening, instead favoring reactive assessment when clinical suspicion arises. Importantly, while the periodicity of screening is not supported by Grade A evidence, several components of auditory care are evidence-based, including the identification of high-risk subgroups (such as children with longer disease duration or poor glycemic control) and the use of more sensitive diagnostic tools like extended high-frequency audiometry and otoacoustic emissions, which are supported by Grade B studies demonstrating that standard pure tone audiometry may miss early diabetic cochlear damage. Thus, current proposals for baseline and follow-up auditory monitoring represent a synthesis of moderate-quality evidence defining risk and test sensitivity, combined with expert consensus addressing gaps where definitive trials are lacking.

## 5. Conclusions

Through a comprehensive review of the literature, a notable link has been established between T1DM and the early onset of auditory dysfunction in children, often affecting both cochlear and retro cochlear pathways. Despite increasing data supporting this association, hearing assessment is not currently embedded within standard clinical guidelines, representing a significant gap in comprehensive monitoring and early detection. A practical and effective approach involves the implementation of periodic audiometric screening, particularly for children with longer disease duration or with persistently elevated HbA1c levels. To clarify the clinical application of these findings, we provide specific indications for when to utilize each diagnostic modality in the pediatric T1DM population.

PTA with EHF testing should be used primarily for routine surveillance in cooperative, older children, typically those aged four to five years and above. Evidence from studies shows that standard audiometry frequently fails to detect early diabetic-related cochlear damage; therefore, clinicians should specifically request EHF testing in the 9–16 kHz range. The pediatric T1DM population with a mean EHF hearing threshold exceeding 15 dB HL requires close monitoring, as EHF audiometry is the most sensitive behavioral method for detecting subclinical cochlear involvement before communication frequencies are affected.

TEOAE, DPOAE, OAEs should be used primarily for screening cochlear microangiopathy in infants, toddlers, or uncooperative children for whom behavioral audiometric testing is unreliable. From an evidence-based perspective, TEOAEs provide an objective and frequency-specific assessment of outer hair cell function. Studies demonstrated reduced TEOAE amplitudes in children with T1DM, reflecting early cochlear dysfunction that often precedes detectable threshold shifts on pure tone audiometry. However, an important limitation is that a “pass” result on OAE testing does not exclude neural hearing loss or auditory neuropathy; OAEs confirm intact outer hair cell function but cannot detect retrocochlear pathology.

ABR testing should be used when diabetic auditory neuropathy or auditory neuropathy spectrum disorder is suspected. Evidence shows that school-aged T1DM mellitus may exhibit prolonged auditory brainstem conduction times and impaired speech perception in noise despite normal findings on standard audiometry. Clinically, ABR is indicated when there is a discrepancy between reported symptoms—such as difficulty hearing in noisy classroom environments—and normal results on OAE and pure tone audiometry. Prolongation of wave I latency or I–V interpeak intervals on ABR may signal early auditory neuropathy that routine screening methods fail to detect.

Further large-scale, longitudinal studies are needed to clarify the pathophysiological mechanisms, establish screening protocols, and evaluate whether interventions—such as improved glycemic control—can preserve auditory function. Randomized controlled trials and the development of sensitive, practical tools for early detection are essential. Until such evidence is available, a proactive, interdisciplinary approach is critical to protect the health, cognitive development, and academic outcomes of pediatric patients with T1DM.

Key summary bullet points:

– Type 1 diabetes in children may lead to auditory dysfunction.
– Hearing impairments associated with diabetes in children are often subclinical.
– Longer duration of diabetes is associated with an increased risk of hearing impairments.
– Impaired hearing may lead to developmental complications in children.

## Figures and Tables

**Table 1:** Summary of key studies and findings.

Author (Year)	*n* (T1DM/Control)	Age (Years)	Hearing Test Used	Key Findings
Treviño-González (2015) [14]	84/0	6–18	Pure-tone audiometry (PTA), Speech audiometry	SNHL prevalence of 14.3%; risk increases significantly with T1DM duration > 5 years (*p* = 0.011). Most deficits were mild, bilateral, and affected high frequencies (8000 Hz).
Yorulmaz et al. (2024) [13]	26/0 *	2–18	PTA (125–8000 Hz), Tympanometry	T1DM duration ≥ 5 years significantly increased thresholds at 4000 and 8000 Hz (*p* < 0.05).
Lieu et al. (2010) [19]	74 matched pairs, total *n* = 148	6–12	Oral and Written Language Scales (OWLS)	Children with unilateral hearing loss (UHL) showed significantly lower scores than healthy siblings in language comprehension (91 vs. 98, *p* = 0.003) and oral expression (94 vs. 101, *p* = 0.007).
Braite et al. (2023) [29]	50/51	7–18	BAEP, event-related potentials (P300)	Significantly higher frequency of memory (*p* = 0.002) and attention (*p* = 0.021) difficulties, associated with prolonged BAEP wave III and V latencies. Poor metabolic control correlated with abnormal BAEP results.
Mohammad et al. (2018) [30]	65/130	10.3 ± 1.7	PTA, ABR, DPOAE (Distortion Product Otoacoustic Emissions)	Mean HbA1c was 7.8 ± 1.6%; 49.2% of children exceeded the glycemic target (>7.5%). Significantly prolonged wave V latency in ABR (*p* < 0.001)

* Control group consisted of T1DM patients with duration < 5 years.

## Data Availability

No new data were created or analyzed in this study.

## Abbreviations

ABR	Brainstem Auditory Evoked Response
ADA	American Diabetes Association
BAEP	Brainstem Auditory Evoked Potentials
CAEPs	Cortical Auditory Evoked Potentials
CI	Confidence Interval
DPOAE	Distortion Product Otoacoustic Emissions
EHF	Extended High-Frequency
HbA1c	Glycated Hemoglobin (Hemoglobin A1c)
HL	Hearing Loss
ISPAD	International Society for Pediatric and Adolescent Diabetes
OAEs	Otoacoustic Emissions
OWLS	Oral and Written Language Scales
SNHL	Sensorineural Hearing Loss
PTA	pure-tone audiometry
T1DM	Type 1 Diabetes Mellitus
TEOAEs	Transient Evoked Otoacoustic Emissions
UHL	Unilateral Hearing Loss
WHO	World Health Organization
